# Immunization against respiratory viruses among dental surgeons: high personal coverage, low involvement in vaccine promotion

**DOI:** 10.1016/j.bjid.2026.105820

**Published:** 2026-05-16

**Authors:** Rodrigo Matheus, Klinger Soares Faico-Filho, Nancy Bellei

**Affiliations:** a Universidade Federal de São Paulo (UNIFESP), Escola Paulista de Medicina (EPM), Laboratório de Virologia, Departamento de Medicina, Divisão de Doenças Infecciosas, São Paulo, SP, Brazil; b Universidade Federal de São Paulo (UNIFESP), Escola Paulista de Medicina (EPM), Departamento de Medicina, Disciplina de Clínica Médica e Medicina Laboratorial, São Paulo, SP, Brazil

**Keywords:** Dentists, Vaccination, Influenza vaccines, COVID-19 vaccines

## Abstract

This study evaluated self-reported immunization coverage against respiratory viruses among dental surgeons and analyzed whether adherence was associated with vaccine-N promotion behaviors in clinical practice. A cross-sectional online survey was conducted between January and April 2025 among dentists registered with the São Paulo Regional Council of Dentistry (CROSP). Of approximately 98,000 dentists, 604 (≈0.6 %) returned responses. Information on sociodemographic characteristics, vaccination status for COVID-19, influenza, and hepatitis B, and professional behaviors related to vaccine promotion was collected through a self-administered questionnaire, without verification of immunization cards. Among 604 participants, adherence was high for COVID-19 primary vaccination (95.2 %), at least one booster dose (90.9 %), and hepatitis B (81.0 %), whereas annual influenza vaccination fell below the ≥ 90 % threshold used to define high coverage (61.1 %). Professional behaviors were less frequent: 17.7 % routinely asked patients about vaccination, 36.2 % actively recommended vaccines, and 17.3 % verified staff vaccination. Questioning patients was positively associated with recommending vaccines (ρ = 0.248; *p* < 0.001) and remained independently associated with it in multivariable analysis (adjusted OR = 4.02; 95 % CI 2.53‒6.40; *p* < 0.001). Coverage was heterogeneous and few professionals translated adherence into vaccine-promotion practices. Integrating vaccine assessment into dental anamnesis and strengthening policies for staff vaccination are opportunities to align dental practice with public health goals.

Vaccination remains a cornerstone of infectious disease prevention and one of the most cost-effective public health strategies worldwide. In healthcare environments, the immunization of professionals plays a dual role: protecting both the worker and the patient. In dentistry, daily procedures such as scaling, polishing, and restorative work generate aerosols capable of transporting respiratory pathogens across the clinical environment.^1^, ^2^, ^3^ These particles may carry microorganisms such as influenza virus, SARS-CoV-2, or Respiratory Syncytial Virus (RSV), representing an occupational risk. Although protective barriers, suction devices, and ventilation systems reduce dispersion, none fully eliminate risk, reinforcing vaccination as an essential biosafety tool.

Beyond personal protection, dental surgeons are expected to act as agents of health promotion, advising patients and maintaining up-to-date team vaccination. The dental office is an environment of continuous interpersonal contact and high trust, which makes the professional's behavior a potential model of preventive attitude. Personal adherence to vaccination may therefore influence patients' and staff's perception of vaccine importance, although this hypothesis requires empirical confirmation. Previous international studies have explored the willingness of dental professionals to engage in vaccine promotion or administration.^4^ and more recent evidence has documented heterogeneous vaccine confidence and advocacy among oral health providers worldwide.^5^, ^6^, ^7^, ^8^ Brazilian data specifically addressing vaccine-promotion behaviors among dental surgeons remain scarce, which reinforces the relevance of national investigations such as the present one.

To address this gap, we conducted a cross-sectional online survey targeting dentists registered in the São Paulo Regional Council of Dentistry (CROSP), the largest regional dental council in Brazil (approximately 98,000 active registrations at the time of the survey). The sampling frame was therefore the entire CROSP population, and the 604 valid responses represent a convenience sample (≈0.6 % of the target population) recruited through institutional electronic mailing. The survey, distributed between January and April 2025, investigated vaccination coverage and three key behaviors related to vaccine promotion: 1) Questioning patients about their vaccination status, operationally defined as self-reporting “often” or “always” addressing immunization during anamnesis (vs. “rarely” or “never”); 2) Actively recommending vaccines, operationally defined as self-reporting “often” or “always” advising patients and/or staff to update their immunization schedule; and 3) Verifying the vaccination status of team members, operationally defined as self-reporting routine documentary verification of staff immunization records. Sociodemographic data such as sex, age, and years since graduation were collected, along with information on professional practice sector (public, private, or mixed). Because the instrument was self-administered and did not require presentation of vaccination cards, responses are subject to recall and social-desirability bias, and coverage figures should be interpreted as self-reported estimates rather than documented coverage. Data analysis included descriptive statistics, Chi-Square tests, Spearman correlations, and multivariable logistic regression models to explore independent associations. The study was approved by the Research Ethics Committee of the Universidade Federal de São Paulo (CEP/UNIFESP; Opinion n°7.282.957; CAAE 82390124.7.0000.5505; December 10, 2024). Participation was voluntary, and informed consent was obtained electronically.

Among 604 valid responses, 63.7 % were women, mean age 41.4-years (SD = 11.7). The mean time since graduation was 14.5-years (SD = 15.4), with 46.5 % of respondents aged 35‒50 years. Most worked in private practice (59.1 %), followed by mixed (20.7 %) and public practice (20.2 %). Self-reported coverage was heterogeneous across vaccines (Table 1): 95.2 % completed the COVID-19 primary vaccination series and 90.9 % had received at least one booster dose (referring to the 2023 and 2024 annual COVID-19 campaigns of the Brazilian National Immunization Program). Adherence to hepatitis-B vaccination (three doses) reached 81.0 %, consistent with occupational health recommendations. Annual influenza vaccination (referring to the 2024 seasonal campaign) showed the lowest adherence (61.1 %), below the ≥ 90 % threshold conventionally used to characterize high coverage. This drop may reflect the optional nature of annual influenza campaigns in the private sector and a perception of lower risk compared with COVID-19, although the present design does not allow causal inference.

**Table 1:** Self-reported vaccination coverage among dental surgeons registered with CROSP (*n* = 604), 2025.

Vaccine / Series	n	Total	%
COVID-19 primary vaccination (≥ 2 doses)	575	604	95.2
COVID-19 booster dose(s) (2023‒2024 campaigns)	549	604	90.9
Annual influenza vaccination (2024 campaign)	369	604	61.1
Hepatitis B vaccination (complete 3-dose series)	489	604	81.0

Professional behaviors directed at vaccine promotion were considerably less frequent than personal adherence (Table 2). Only 17.7 % routinely addressed vaccination during dental anamnesis, 36.2 % actively recommended vaccines to patients, and 17.3 % verified staff vaccination. Approximately one-third (33.8 %) never verified the vaccination status of their team. These findings suggest a gap between individual adherence and the systematic incorporation of immunization into clinical routine; however, because the study is cross-sectional and behaviors were self-reported, this gap should be interpreted as a descriptive observation rather than as evidence of a behavioral mechanism.

**Table 2:** Self-reported vaccine-promotion behaviors among dental surgeons registered with CROSP (*n* = 604), 2025.

Professional behavior	n	Total	%
Frequently questions patients about vaccination status	108	604	17.7
Actively recommends vaccines to patients and/or staff	220	604	36.2
Frequently verifies staff vaccination status	107	604	17.3
Never verifies staff vaccination status	209	604	33.8

Bivariate analyses indicated that professionals aged 35‒50 years were more likely to question patients about vaccination (χ^2^ = 8.380; *p* = 0.015). This age-group difference is hypothesis-generating and may reflect a balance between clinical experience and recent exposure to continuing education, but it cannot be confirmed with the present data. Questioning patients about vaccination showed a positive correlation with active vaccine recommendation (ρ = 0.248; *p* < 0.001), suggesting that these two behaviors tend to co-occur in clinical routine. No significant correlation was observed between questioning patients and verifying staff vaccination (ρ = −0.004; *p* = 0.924).

Multivariable logistic regression confirmed an independent association between questioning patients and actively recommending vaccines (adjusted OR = 4.02; 95 % CI 2.53‒6.40; *p* < 0.001). Other variables such as sex, age, or years since graduation were not independently associated with vaccine-promotion behaviors. However, the regression model was limited to sociodemographic and practice-sector variables and did not include potentially important confounders such as formal training in public health, access to vaccination guidelines, institutional vaccination policies, or workplace biosafety culture ‒ factors that could substantially influence both outcomes. The observed associations should therefore be viewed as preliminary, and further studies incorporating these variables are warranted. No independent associations were identified for the behavior of verifying team vaccination, which remains an undervalued dimension of biosafety in dental environments according to the present sample.

Stratified analyses by age revealed that professionals under 35-years had lower rates of patient vaccination questioning (14.0 %) compared to those aged 35‒50 years (21.7 %) and over 50-years (11.5 %). The lower rate among the oldest group might reflect more stable clinical routines with less frequent adoption of new preventive practices, although this interpretation is speculative. Public-sector dentists exhibited a slightly higher frequency of vaccine recommendation (50.0 %) than those in private (42.3 %) or mixed practices (40.8 %), though the difference was not statistically significant (*p* = 0.290). This pattern is consistent with the hypothesis that institutional policies and continuing education programs in the public health system favor vaccine advocacy, but the current data do not allow this hypothesis to be tested directly.

The observed gap between individual immunization and professional engagement represents a missed opportunity for integrating public health promotion within dentistry. Recent international literature has described similar patterns among oral health providers and has highlighted the potential role of dentists as vaccine advocates.^5^, ^6^, ^7^, ^8^ Incorporating vaccine-related questions into standard anamnesis, verifying team immunization records, and using reminder systems within dental software are feasible strategies that could plausibly strengthen vaccination culture in dental practices. Educational institutions and professional councils could also include vaccination topics in continuing education curricula and ethical guidelines.

From a public health perspective, the post-pandemic re-emergence of respiratory threats such as RSV and influenza variants^9^^,^^10^ reinforces the relevance of multidisciplinary collaboration between dentistry and preventive medicine. Institutional initiatives, including annual audits and workplace vaccination campaigns, may contribute to bridging the gap between awareness and action. Encouraging dentists to view vaccination as part of patient safety and infection control, rather than an isolated health behavior, is a plausible avenue to enhance the resilience of healthcare systems against future respiratory outbreaks.

This study has several limitations that should be considered when interpreting the findings. First, the sample corresponds to a small fraction of the CROSP-registered population (≈0.6 %) and was obtained through voluntary participation, which may introduce selection bias toward professionals more engaged with immunization topics. Second, all vaccination and behavioral variables were self-reported and were not cross-checked against immunization cards or institutional records, so recall and social-desirability bias cannot be excluded. Third, the cross-sectional design precludes causal inference between questioning patients and recommending vaccines. Fourth, the regression model did not capture potential confounders such as public-health training, access to vaccination guidelines, and institutional policies. Fifth, the study was restricted to the State of São Paulo and may not reflect the reality of other Brazilian regions. We were unable to identify published Brazilian studies specifically assessing vaccine-promotion behaviors among dental surgeons, which itself underscores the need for further national research.

In conclusion, coverage was heterogeneous across vaccines ‒ ranging from 61.1 % for influenza to 95.2 % for COVID-19 primary vaccination ‒ and only COVID-19 reached the ≥90 % threshold conventionally used to characterize high coverage. Engagement in vaccination promotion was consistently lower than personal adherence. Incorporating structured vaccine assessments into dental anamnesis and systematically verifying staff vaccination are plausible strategies to foster a safer and more preventive clinical culture. These measures may contribute to protecting professionals and patients and to aligning dental practice with the broader goals of public health across the Americas.

## Data availability

The data that support the findings of this study are available from the corresponding author upon reasonable request.

## Conflicts of interest

The authors declare no conflicts of interest.
